# Pathway-Based Polygenic Risk Scores for Schizophrenia and Associations With Reported Psychotic-like Experiences and Neuroimaging Phenotypes in the UK Biobank

**DOI:** 10.1016/j.bpsgos.2023.03.004

**Published:** 2023-03-25

**Authors:** Miruna C. Barbu, Maria Viejo-Romero, Gladi Thng, Mark J. Adams, Katie Marwick, Seth G.N. Grant, Andrew M. McIntosh, Stephen M. Lawrie, Heather C. Whalley

**Affiliations:** a Division of Psychiatry, The University of Edinburgh, Royal Edinburgh Hospital, Edinburgh, Scotland, United Kingdom; b Genes to Cognition Program, Centre for Clinical Brain Sciences, University of Edinburgh, Edinburgh, Scotland, United Kingdom

**Keywords:** Biological pathway, Neuroimaging, Pathway-based polygenic risk scores, Schizophrenia, Synaptic processes, UK Biobank

## Abstract

**Background:**

Schizophrenia is a heritable psychiatric disorder with a polygenic architecture. Genome-wide association studies have reported that an increasing number of risk-associated variants and polygenic risk scores (PRSs) explain 17% of the variance in the disorder. Substantial heterogeneity exists in the effect of these variants, and aggregating them based on biologically relevant functions may provide mechanistic insight into the disorder.

**Methods:**

Using the largest schizophrenia genome-wide association study conducted to date, we associated PRSs based on 5 gene sets previously found to contribute to schizophrenia pathophysiology—postsynaptic density of excitatory synapses, postsynaptic membrane, dendritic spine, axon, and histone H3-K4 methylation—along with respective whole-genome PRSs, with neuroimaging (*n* > 29,000) and reported psychotic-like experiences (*n* > 119,000) variables in healthy UK Biobank subjects.

**Results:**

Several variables were significantly associated with the axon gene-set (psychotic-like communications, parahippocampal gyrus volume, fractional anisotropy thalamic radiations, and fractional anisotropy posterior thalamic radiations (β range −0.016 to 0.0916, false discovery rate–corrected *p* [*p*_FDR_] ≤ .05), postsynaptic density gene-set (psychotic-like experiences distress, global surface area, and cingulate lobe surface area [β range −0.014 to 0.0588, *p*_FDR_ ≤ .05]), and histone gene set (entorhinal surface area: β = −0.016, *p*_FDR_ = .035). From these, whole-genome PRSs were significantly associated with psychotic-like communications (β = 0.2218, *p*_FDR_ = 1.34 × 10^−7^), distress (β = 0.1943, *p*_FDR_ = 7.28 × 10^−16^), and fractional anisotropy thalamic radiations (β = −0.0143, *p*_FDR_ = .036). Permutation analysis revealed that these associations were not due to chance.

**Conclusions:**

Our results indicate that genetic variation in 3 gene sets relevant to schizophrenia may confer risk for the disorder through effects on previously implicated neuroimaging variables. Because associations were stronger overall for whole-genome PRSs, findings here highlight that selection of biologically relevant variants is not yet sufficient to address the heterogeneity of the disorder.

Schizophrenia is a psychiatric disorder characterized by positive and negative symptoms, as well as marked cognitive impairment (1). Schizophrenia is thought to result from a complex combination of genetic and environmental factors, and its heritability has been estimated at 80% (2).

Genome-wide association studies (GWASs) have reported increasing numbers of genomic loci associated with schizophrenia, lending support to the contribution of common genetic variants to the pathophysiology of schizophrenia (3,4). Genome-wide polygenic risk scores (PRSs) calculated from GWASs explain ∼17% (Nagelkerke’s *R*^2^) of the variance in schizophrenia (4). However, substantial heterogeneity exists in the effect of risk variants, and genome-wide approaches may not be sufficient for patient stratification in downstream analyses. Mechanistic insight may be derived from GWASs, including the identification of biologically relevant gene sets in which risk variants are aggregated. For instance, the Psychiatric Genomics Consortium (PGC) identified a number of pathways specific to schizophrenia, major depressive disorder, and bipolar disorder but also pathways common across the three, including histone methylation, immune, and neuronal signaling pathways (5).

Combining the predictive power of PRSs with findings from pathway analysis by examining genetic variation within biologically relevant gene sets may address the inherent heterogeneity in schizophrenia and provide additional mechanistic insight by detecting associations with biologically informative traits. This methodology has been applied to a number of schizophrenia-relevant phenotypes. Rampino *et al.* (6) found that schizophrenia PRSs calculated based on single nucleotide polymorphisms (SNPs) implicated in glutamatergic signaling were associated with attention, a cognitive process known to be impaired in schizophrenia. Yao *et al* (7) found that PRSs calculated based on neural microRNA-137 (*MIR137*) explained a disproportionately larger schizophrenia risk variance than genomic control PRSs when accounting for gene-set size (∼2% *MIR137* = ∼1000 genes; ∼10%, whole-genome [WG] = ∼20,000 genes). Therefore, it is possible to interrogate genetic risk aggregated to biologically relevant gene sets to gain insight into the association between aggregated genetic risk and disorder-specific traits of interest.

Previous evidence has also shown associations of schizophrenia PRSs with disruptions in white matter microstructure and global and regional brain volumes, although results have been inconsistent (8,9). As such, it has been suggested that biologically relevant gene sets may reveal stronger associations with neuroimaging phenotypes, as demonstrated recently (10). Grama *et al.* (10) investigated whether behavior- and neuronal-related gene sets, previously implicated in schizophrenia, were associated with subcortical volumes. They found that one gene set, “abnormal behavior,” was associated with right thalamic volume, and this association was robust across different *p*-value thresholds (10). This methodology can be applied to other psychiatric disorders. For instance, PRSs calculated for a previously established biological pathway (NETRIN1) in relation to major depressive disorder were associated with relevant neuroimaging phenotypes, shedding light on links between biology and neuroimaging (11). This indicates that meaningful associations with traits of interest may be revealed when applying genomic methods that address relevant parts of the genome.

Based on this evidence, we hypothesized that schizophrenia PRSs aggregated in biologically relevant pathways previously shown to play a role in schizophrenia would be associated with structural neuroimaging and reported psychotic-like experience (PLE) phenotypes in a sample of adults in the general population. Identifying associations with specific neuroimaging phenotypes may provide an opportunity to disentangle the heterogeneity of the disorder, in terms of both genetic risk and inconsistent previous neuroimaging findings. Therefore, we selected 5 gene sets previously identified by the PGC (5): postsynaptic density (PSD), postsynaptic membrane (PSM), dendritic spine, axon, and histone H3-K4 methylation. These 5 cellular components and biological processes have been associated with schizophrenia in a number of studies investigating human and animal models (12, 13, 14). We calculated PRSs for each gene-set–specific set of SNPs and for SNPs excluded from the gene sets for paired comparisons (gene-set SNPs vs. WG minus gene-set SNP PRSs). Then, we tested their association with brain structural phenotypes (cortical volume, thickness, and surface area; white matter microstructure indexed by fractional anisotropy [FA] and mean diffusivity [MD]; and subcortical volumes) and reported PLEs (Mental Health Questionnaire [MHQ]) in the UK Biobank (UKB), utilizing the most up-to-date genetic, mental health, and imaging data. We hypothesized distinctive roles in the pathophysiology of schizophrenia for the different biologically relevant pathways tested, after adjustment for WG PRSs (excluding SNPs in each gene set), highlighting important mechanistic processes underlying the different phenotypes associated with schizophrenia. We expected this methodology to partly address the heterogeneity in schizophrenia through the identification of biologically relevant mechanisms.

## Methods and Materials

### Study Population

The UKB comprises of 502,411 community-dwelling individuals recruited between 2006 and 2010 in the United Kingdom (https://biobank.ctsu.ox.ac.uk/crystal/field.cgi?id=200) (15). The UKB received ethical approval from the research ethics committee (Reference 11/NW/0382). This study was approved by the UKB Access Committee (Project Nos. 4844 and 16124). Written informed consent was obtained from all participants. This study was conducted using the latest release of UKB neuroimaging data (*n* = 29,791 cortical, *n* = 29,536 subcortical, *n* = 27,917 white matter) and *n* = 119,947 with MHQ data. We excluded individuals with a diagnosis of schizophrenia as indicated by ICD-10 (F20, https://biobank.ndph.ox.ac.uk/showcase/field.cgi?id=41270) due to the small proportion of diagnosed individuals (*n* = 989) and the fact that our analyses focused on associations in the general population.

### Gene-Set Selection

The top 5 gene sets associated with schizophrenia were selected from The Network and Pathway Analysis Subgroup of the PGC (5) and identified on Gene Ontology (GO) by searching for each pathway’s GO identifier in the PGC study (5) [see also (16)]: PSD (GO:0014069), PSM (GO:0045211), dendritic spine (GO:0043197), axon (GO:0030424), and histone H3-K4 methylation (GO:0051568). The gene sets were selected based on their robust associations with schizophrenia in this and previous studies (5,13,17,18). Further details on gene sets are included in the Supplement and Supplementary Excel File 1.

### Genotyping, SNP Annotation, and PRS Profiling

Genotyping of 488,000 blood samples from UKB participants was carried out using the UK BiLEVE (https://biobank.ctsu.ox.ac.uk/crystal/refer.cgi%3fid%3d149600) or UKB Axiom (https://biobank.ctsu.ox.ac.uk/crystal/refer.cgi%3fid%3d149601) arrays (19). Further information on genotyping procedures and quality control are provided in https://biobank.ctsu.ox.ac.uk/crystal/crystal/docs/genotyping_qc.pdf and in Bycroft *et al.* (19). Genetic data from both the base and target datasets were annotated in reference to human genome build 19.

We used SNPs from the largest available GWAS of schizophrenia (*N* = 320,404, *n* = 76,755 cases), which was carried out by Trubetskoy *et al.* (4). They identified schizophrenia associations with common variants at 287 distinct loci, with PRSs explaining ∼17% of the variance in a European ancestry target sample (4). The GWAS sample utilized here did not include any individuals in UKB. The full SNP quality control protocol is detailed in Supplement and was based on Choi *et al.* (20) (https://choishingwan.github.io/PRS-Tutorial/base/). In addition, we ensured that our sample consisted of unrelated, White British participants with no overlap with the PGC sample (21). The final genetic sample consisted of *N* = 365,125 participants and *N* = 5,974,990 SNPs, which was further reduced when combining with the reported-PLEs and imaging samples (see below).

Following functional annotation (22) (see the Supplement), SNPs located within each gene set were extracted. See Table S1 in the Supplement and Supplementary Excel File 1 for the number of genes and SNPs within each gene set. From these lists, gene-set PRSs were computed for each individual in the UKB (see the Supplement) using PRSice (23) at 5 *p*-value thresholds (.01, .05, .1, .5, 1). Each gene-set PRS had its respective WG PRS (i.e., each gene-set SNP list was input as an exclusion flag to create WG PRSs that did not include the specific gene sets). The primary analysis in this manuscript comprises SNPs that met a significance level of .1 (4). Analyses at other thresholds are included in Tables S5 to S11 in Supplementary Excel File 2.

### Phenotypes

#### Psychotic-like Experiences

An MHQ was sent to participants who provided an email address between July 2016 and July 2017 (*n* = ∼157,000). This included 4 unusual and psychotic experience items, the dichotomized responses to which were used to create the 4 lifetime PLEs utilized in this study, which will be referred to as “conspiracies,” “communications,” “voices,” and “visions” based on their clinical significance (see the Supplement for the list of questions). We also examined distress by selecting individuals who reported PLEs as distressing (as opposed to neutral or positive) and those who reported no PLEs to determine associations with experiencing stress (24, 25, 26), which will be referred to as “distress.” Frequencies for all items are noted in Table 1.

**Table 1:** Demographic Characteristics of the Reported-PLEs Sample

	MHQ Sample
Age, Years, Mean (SD)	56.09 (7.69)
Sex, Female, *n* (%)	67,432 (56%)
MHQ—Heard Unreal Voice, *n* (%)	1918 (1.6%)
MHQ—Seen Unreal Vision, *n* (%)	3696 (3%)
MHQ—Believed in Unreal Conspiracy, *n* (%)	724 (0.60%)
MHQ—Believed in Unreal Communications, *n* (%)	652 (0.54%)
Distressing PLEs, *n* (%)	2046 (1.76%)

MHQ, Mental Health Questionnaire; PLE, psychotic-like experience.

#### Neuroimaging Phenotypes

A brain magnetic resonance imaging (MRI) scan was conducted for a subset of participants (27,28), and imaging-derived phenotypes of T1-weighted and diffusion MRI images were used in this study. MRI acquisition, preprocessing, and quality control protocols can be found in the Supplement. Individuals with global values >3 SD from the sample mean, as ascertained by conducting principal component (PC) analysis on each modality’s imaging variables to derive a global value, were excluded (range *n* excluded based on imaging modality = 105–232 participants) (distribution plots created using “ggplot” in *R* are shown in Figure S1 in the Supplement) (29,30). A list of neuroimaging phenotypes investigated is included in Table S2 in the Supplement.

Fifteen white matter tracts (3 unilateral) from 2 diffusion scalars, FA and MD, were utilized (https://biobank.ndph.ox.ac.uk/showcase/label.cgi?id=135). Cortical regions of interest (ROIs) were identified using Desikan-Killiany-Tourville parcellation in FreeSurfer (https://surfer.nmr.mgh.harvard.edu/), resulting in 31 cortical structures per hemisphere for cortical thickness, surface area (SA), and volume (31,32) (https://biobank.ndph.ox.ac.uk/showcase/label.cgi?id=196). Eight subcortical gray matter ROIs per hemisphere were also identified (33).

For white matter microstructure, we derived global and regional (association and projection fibers, thalamic radiations) measures of FA and MD by conducting PC analysis on sets of tracts and extracting scores of the first unrotated principal component. For cortical ROIs, we derived global and lobar measures by summing up all (global) or lobar-specific ROIs, as in previous studies (34,35). Sample size and descriptive statistics for neuroimaging phenotypes are presented in Table 2.

**Table 2:** Demographic Characteristics of the Neuroimaging Samples

	Neuroimaging Sample
Age, Years, Mean (SD)	63.71 (7.48)
Sex, Female, *n* (%)	
Cortical regions	15,666 (53%)
Subcortical volumes	15,557 (53%)
White matter microstructure	14,745 (53%)
Scan Site, *n*	
Cheadle	24,910
Reading	5064
Newcastle	9968

*n* is different for each variable because outlier exclusion (3 SDs from mean) was applied individually to each phenotype.

### Statistical Analysis

All analyses were conducted using R (version 4.1.0; R Core Team) in a Linux environment. We used linear mixed-effects models (function “lme” in package “nlme”) for bilateral neuroimaging phenotypes, with age, age^2^, sex, 15 genetic PCs, scan site, 3 MRI head position coordinates (lateral brain position X https://biobank.ctsu.ox.ac.uk/crystal/field.cgi?id=25756, transverse brain position Y https://biobank.ctsu.ox.ac.uk/crystal/field.cgi?id=25757, and longitudinal brain position Z https://biobank.ctsu.ox.ac.uk/crystal/field.cgi?id=25757), and genotype array set as covariates. Hemisphere was included as a within-subject covariate in all mixed-effects models. Intracranial volume was included as a covariate for gray matter phenotypes. We used general linear models (function “lm”) for unilateral, regional, and global neuroimaging phenotypes, using the same covariates as above without hemisphere included. Finally, we used logistic regression for dichotomized PLEs, with age, sex, 15 genetic PCs, and genotype array as covariates. All models included gene-set PRS and each gene set’s respective WG (excluding SNPs in each gene set) PRS as predictor variables. False discovery rate (FDR) was used to correct for multiple testing and was applied separately for each neuroimaging and reported-PLEs phenotype and across all *p*-value thresholds (36) (see Table S3 in the Supplement for detailed protocol). Effect sizes from linear models were standardized throughout.

To establish that the effect of gene-set PRSs on neuroimaging and reported-PLEs phenotypes was not due to chance (because the SNP set sizes in all gene sets were much smaller than the WG), permutation analysis was carried out using a method developed by Cabrera *et al.* (37) for all significant associations (see the Supplement).

## Results

All results presented below concern PRSs at a *p*-value threshold < .1. Descriptive statistics for the reported-PLEs and neuroimaging phenotypes are noted in Tables 1 and 2, respectively. Analyses involving other thresholds are included in Tables 5–11 in Supplementary Excel File 2.

### Associations With PLEs

**Figure 1:**
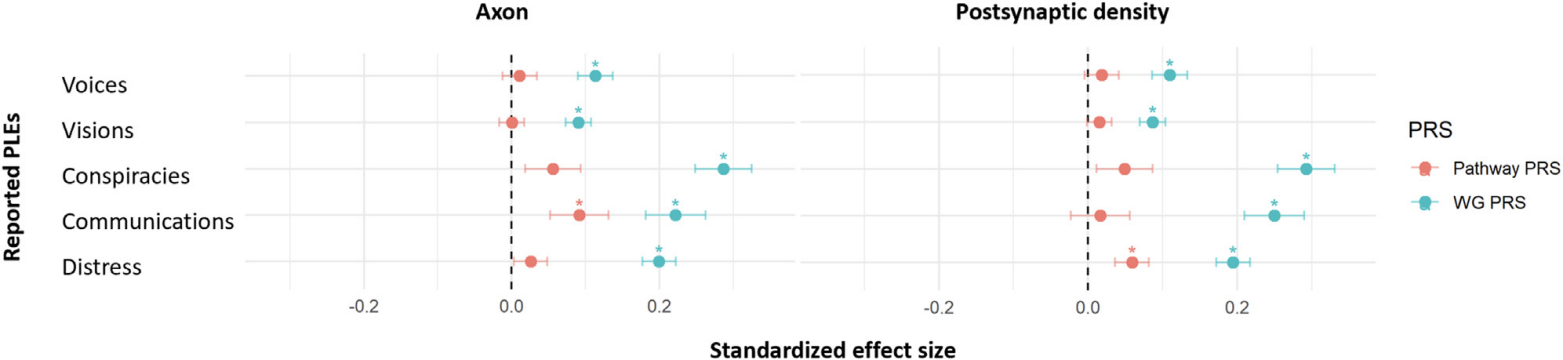
Significant associations between the axon and postsynaptic density gene-set PRSs and reported-PLEs phenotypes. The x-axis indicates standardized effect sizes; ∗ indicates significant associations after correction for multiple comparisons. PLE, psychotic-like experience; PRS, polygenic risk score; WG, whole-genome (excluding single nucleotide polymorphisms in each gene set).

Gene set–specific significant findings and corresponding WG (excluding SNPs in each gene set) results are noted in Figure 1. The axon gene-set PRS was significantly associated with the communications MHQ item (gene-set PRS β = 0.0916, *p*_FDR_ = .04; WG PRS [excluding SNPs in each gene set] β = 0.2218, *p*_FDR_ = 1.34 × 10^−7^), while the PSD gene-set PRS was significantly associated with distress associated with reported PLEs (gene-set PRS β = 0.0588, *p*_FDR_ = .02; WG PRS [excluding SNPs in each gene set] β = 0.1943, *p*_FDR_ = 7.28 × 10^−16^). WG PRSs (excluding SNPs in each gene set) were associated with all 4 MHQ items as well as distress, and effect sizes were of greater magnitude than associations with gene-set PRSs (see Table S5 in Supplementary Excel File 2). Effect sizes for gene set–specific PRSs ranged from 1 × 10^−4^ to 0.092 and for WG PRSs (excluding SNPs in each gene set) ranged from 0.087 to 0.300. Results concerning MHQ items at other *p*-value thresholds are provided in Table S5 in Supplementary Excel File 2.

### Associations With Neuroimaging Phenotypes

#### Gene-Set PRS Associations

Axon gene-set PRSs were associated with parahippocampal gyrus volume (gene-set PRS β = 0.0156, *p*_FDR_ = .03; WG [excluding SNPs in each gene set] PRS β = −0.003, *p*_FDR_ = .833), FA thalamic radiations tract (gene-set PRS [excluding SNPs in each gene set] β = −0.014, *p*_FDR_ = .036; WG PRSs [excluding SNPs in each gene set] β = −0.0143, *p*_FDR_ = .036), and FA posterior thalamic radiations (gene-set PRS β = −0.016, *p*_FDR_ = .048; WG PRSs [excluding SNPs in each gene set] β = −0.011, *p*_FDR_ = .126).

PSD gene-set PRSs were associated with global SA (gene-set PRS β = −0.012, *p*_FDR_ = .034; WG PRSs [excluding SNPs in each gene set] β = −0.003, *p*_FDR_ = .517) and cingulate lobe SA (gene-set PRS β = −0.014, *p*_FDR_ = .04; WG PRSs [excluding SNPs in each gene set] β = 1 × 10^−4^, *p*_FDR_ = .977).

**Figure 2:**
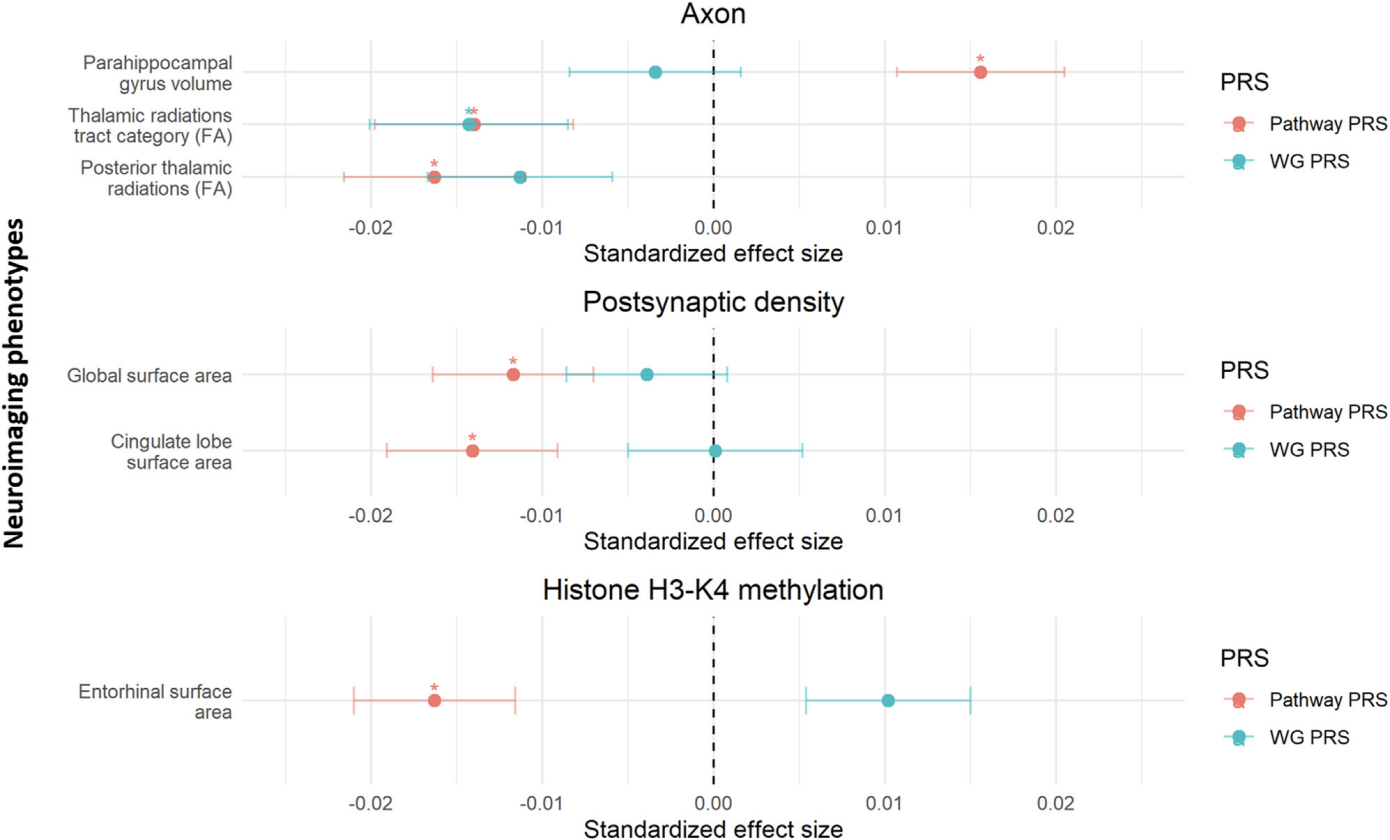
Significant associations between the axon, postsynaptic density, and histone H3-K4 methylation gene-set PRSs and neuroimaging phenotypes. The x-axis indicates standardized effect sizes; ∗ indicates significant associations after correction for multiple comparisons. FA, fractional anisotropy; PRS, polygenic risk score; WG, whole-genome (excluding single nucleotide polymorphisms in each gene set).

**Figure 3:**
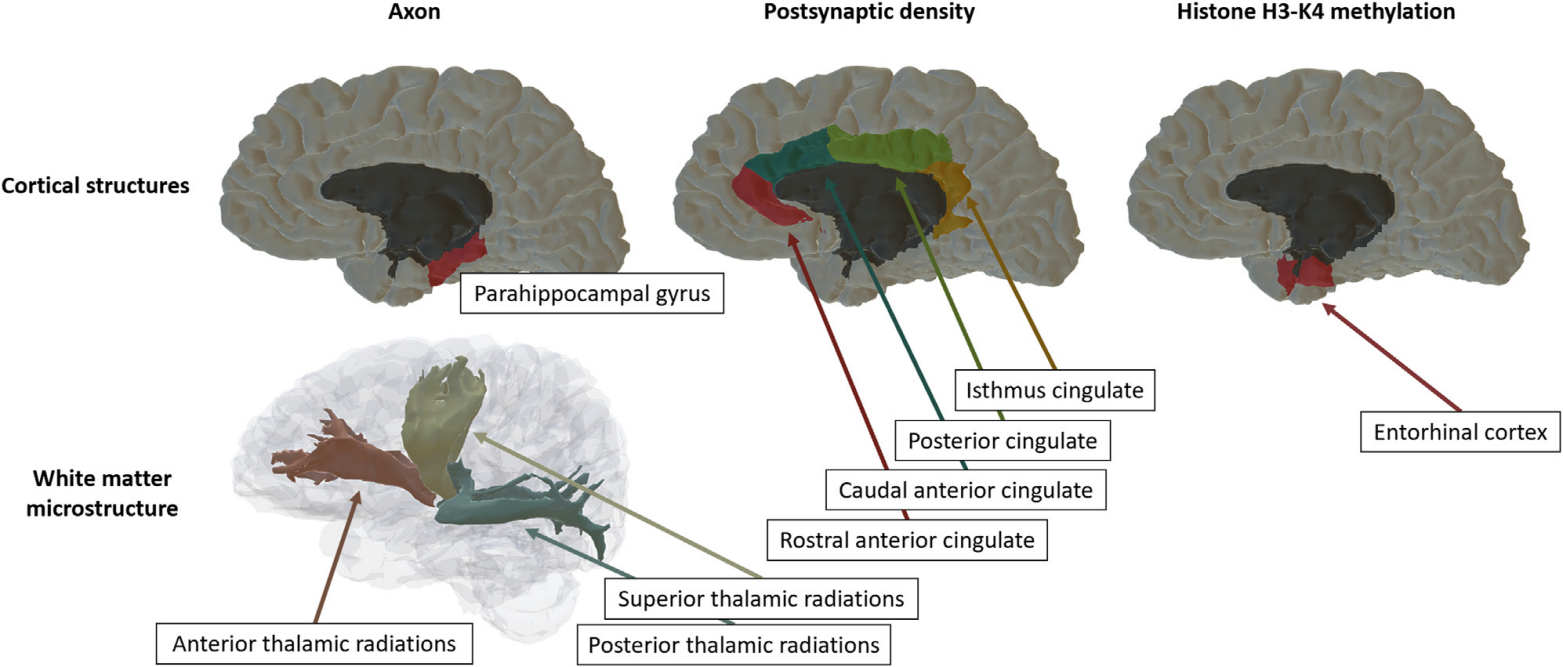
Cortical and white matter microstructure phenotypes that were associated with the axon, postsynaptic density, and histone gene-set polygenic risk scores. The thalamic radiations tract category, comprising the anterior, superior, and posterior thalamic radiations, was associated with the axon gene-set polygenic risk score; the cingulate lobe, comprising caudal anterior, rostral anterior, posterior and isthmus cingulate, was associated with postsynaptic density gene-set polygenic risk scores. Global surface area (i.e., the entire brain’s surface area) was also associated with postsynaptic density, but this is not indicated in the graph above.

Finally, the histone H3-K4 methylation gene-set PRSs were associated with entorhinal SA (gene-set PRS β = −0.016, *p*_FDR_ = .035; WG PRSs [excluding SNPs in each gene set] β = 0.01, *p*_FDR_ = .164). Neuroimaging phenotypes associated with gene-set PRSs are indicated in Figures 2 and 3.

### WG PRS Associations

WG PRSs, irrespective of which SNPs were removed prior to calculating PRSs, were associated with a number of neuroimaging phenotypes indicated in Table S4 in the Supplement and Tables S6–S11 in Supplementary Excel File 2. All standardized beta values are absolute, and ranges are not reported if they are rounded to the same number.

Results are consistent across WG PRSs in which different gene-set SNPs were excluded. FA: Global FA (β range = 0.016–0.019, *p*_FDR_ range = .004–.03), association fibers (β range = 0.018–0.021, *p*_FDR_ range = .003–.019), thalamic radiations (β range = 0.014–0.018, *p*_FDR_ range = .006–.03), cingulate gyrus (β range = 0.014–0.015, *p*_FDR_ range = .014–.034), anterior (β range = 0.016–0.017, *p*_FDR_ range = .019–.038) and posterior (β range = 0.015–0.016, *p*_FDR_ range = .025–.03) thalamic radiations, inferior longitudinal fasciculus (β range = 0.015–0.017, *p*_FDR_ range = .015–.043), and inferior fronto-occipital fasciculus (β range = 0.015–0.016, *p*_FDR_ range = .024–.035).

MD: Global MD (β range = 0.015–0.017, *p*_FDR_ range = .011–.04), association fibers (β range = 0.013–0.014, *p*_FDR_ range = .034–.042), thalamic radiations (β range = 0.014–0.016, *p*_FDR_ range = .011–.04), projection fibers (β range = 0.014, *p*_FDR_ = .04), corticospinal tract (β range = 0.02–0.022, *p*_FDR_ range = .005–.023), anterior (β = 0.015, *p*_FDR_ = .037), superior (β = 0.014, *p*_FDR_ = .043), and posterior (β = 0.013, *p*_FDR_ = .04) thalamic radiations, inferior longitudinal fasciculus (β = 0.015, *p*_FDR_ = .04), and cingulate gyrus (β = 0.015, *p*_FDR_ = .04).

The thalamus (β range = 0.019–0.023, *p*_FDR_ range = 3.6 × 10^−5^–.0009) and accumbens (β range = 0.013−0.015, *p*_FDR_ range = .005–.032) were also significantly associated with WG PRSs (excluding SNPs in each gene set) after FDR correction. Finally, significant cortical regions included volume of the medial orbitofrontal cortex (β range = 0.016–0.019, *p*_FDR_ range = .003–.027) and superior temporal gyrus (β range = 0.018–0.02, *p*_FDR_ range = 8.3 × 10^−4^–.009) and SA of the superior temporal gyrus (β range = 0.015–0.016, *p*_FDR_ range = .022–.04).

### Permutation Analysis

For gene-set PRSs that were significantly associated with reported PLEs or neuroimaging measures, we performed circular genomic permutation analysis. We found that the significant associations with the gene-set PRSs in these phenotypes were not due to chance based on a permutation *p* value that was computed by comparing *t* values from the real associations with *t* values from the permuted associations (Table 3).

**Table 3:** Permutation Results for Gene-Set PRSs Where Significant Associations Were Identified

Gene-Set PRSs	Phenotype	Gene-Set PRS β	Gene-Set PRS *t* Value	Gene-Set PRS Calculated *p* Value
Axon	Communications	0.0916	2.306	.026
	Parahippocampal gyrus volume	0.0156	3.169	.003
	Thalamic radiations (FA)	−0.014	−2.44	.01
	Posterior thalamic radiations (FA)	−0.016	−3.055	.004
Postsynaptic Density	Distress	0.0588	2.618	.01
	Cingulate lobe surface area	−0.014	−2.812	.012
	Global surface area	−0.012	−2.498	.024
Histone H3-K4 Methylation	Entorhinal cortex	−0.016	−3.454	.002

PRS *p*-value threshold = .1.

FA, fractional anisotropy; PRS, polygenic risk score.

## Discussion

In this study, we investigated associations between biologically relevant pathway PRSs and 119,947 reported-PLEs and ∼29,000 neuroimaging phenotypes after adjustment for respective WG PRSs (excluding SNPs in each gene set). We calculated PRSs using the largest, most recent schizophrenia GWAS to date (4). We found significant associations of axon, PSD, and histone H3-K4 methylation gene sets with several cortical regions and white matter tracts, and with reported PLEs, specifically psychotic-like communications and distress associated with a reported PLE. Associations with reported PLEs were stronger for WG PRSs (excluding SNPs in each gene set), while associations with neuroimaging variables were stronger for gene-set PRSs.

The 3 gene sets with significant associations identified here have previously been linked to schizophrenia. The PGC (5) identified pathways implicated in schizophrenia through robust computational analyses by aggregating gene sets from a large number of databases. The most significant genetic contribution to schizophrenia was found in genes encoding proteins located in excitatory synapses, in particular the proteome of the PSD, followed by histone methylation–related processes. The PSD of excitatory synapses comprises protein complexes that assemble glutamate-sensitive neurotransmitter receptors to intracellular proteins (38,39). Postsynaptic networks have long been implicated in schizophrenia (40, 41, 42) because they play a role in cognition and synaptic plasticity, features known to be disrupted in schizophrenia (13,43). In addition, both postsynaptic and presynaptic networks were uncovered through gene prioritization in the latest schizophrenia GWAS, highlighting the consistency of associations with this pathway and further rationale to investigate the pathway here (4).

We identified associations between the PSD gene set and decreased global and cingulate lobe SA, as well as higher PLE-associated distress. The cingulate lobe is part of the limbic system and comprises 4 cortical regions: rostral, caudal, posterior, and isthmus cingulate. The region is involved in behavior, emotion regulation, and cognitive processes including memory, attention, and motivation, all of which are disrupted in schizophrenia (44). Volumes in this region were reduced in previous studies investigating their association with schizophrenia, and a systematic review concluded that hypoactivity of the cingulate cortex underlies the manifestation of negative symptoms in many patients, although the studies analyzed provided inconsistent results (44,45). Interestingly, WG PRSs have not shown associations with cingulate or global SA in previous studies (46,47). Associations identified here indicate that narrowing the genome to a biologically relevant gene set may reveal associations that are not observed genome-wide.

The axon gene set, a cellular component that conducts electrical signals to presynaptic boutons that store and release neurotransmitters, has also been found to play an important role in schizophrenia. Specifically, disruption in axon guidance and axonal growth have been associated with increased schizophrenia risk in both human and mouse models (48,49). A recent study showed that individuals at high genetic risk for schizophrenia had hemispherical asymmetry in whole-brain structural networks, indicating that genetic susceptibility to schizophrenia modulated white matter network abnormalities. In addition, gene-set enrichment analysis found that genes participating in the PRS threshold used were involved in multiple relevant pathways, including axonal growth and axon guidance (18). Therefore, the axon gene set is a highly relevant route of investigation in the context of schizophrenia. Here, the axon gene set was associated with thalamic radiations, white matter microstructural tracts that link the thalamus to the rest of the cerebral cortex (50), and volume of the parahippocampal gyrus, a cortical region that plays a role in memory processes such as encoding and retrieval (51). Both neuroimaging phenotypes have been implicated in schizophrenia (52) and have recently been associated with state anhedonia and PRSs for anhedonia, a core negative symptom of schizophrenia (53). An opposite directionality of effect for WG PRSs and gene-set PRSs on parahippocampal gyrus volume could denote differential pathway-specific action on this region. The findings here indicate that genes conferring risk for schizophrenia that are aggregated in the axon gene set are strengthening evidence for brain structural regions that have already been implicated in schizophrenia psychopathology.

Finally, the histone H3-K4 methylation gene set was associated with entorhinal SA here. Histone H3-K4 methylation involves the modification of histone H3 by the addition of one or more methyl groups to lysine at position 4 of the histone. Histones, and specifically H3, are used to package DNA, and modifications lead to changes in gene expression (54). Epigenetic processes, including histone and DNA methylation, have been associated with schizophrenia through candidate gene regulation (HDAC1, GAD67) and epigenome-wide studies, lending support to the investigation of epigenetic modifications in schizophrenia (55, 56, 57). A key feature of schizophrenia is that it has its age of onset in young adulthood, and the transcriptional regulation of schizophrenia risk genes encoding synaptic proteins occurs at the age of onset, potentially through mechanisms involving H3-K4 methylation (42).

The entorhinal cortex plays a role in the integration of multisensory information between cortical and subcortical structures. It is strongly connected to the hippocampus and is involved in memory processes such as formation and consolidation (58). In studies investigating a neurodevelopmental animal model of schizophrenia, lesions in the entorhinal cortex, created early in the developmental period, were associated with enhanced mesolimbic dopamine release at a later time point, which was expressed through increased locomotor activity. This indicates that structural disruptions in this area are relevant for schizophrenia-like presentations (59). Interestingly, in an animal model, H3-K4 methylation was found to be upregulated in the hippocampus 1 hour after induction of contextual fear conditioning, a memory-related task that aims to rapidly create context-related fear memories (17). Although not directly related to the entorhinal cortex, this finding indicates that H3-K4 methylation may be a useful candidate in the investigation of some schizophrenia-related symptoms in a subcortical structure closely linked to the entorhinal region. Lastly, in a recent human study investigating cortical thickness, SA, and folding index of the entorhinal cortex, Schultz *et al.* (58) uncovered a link between left SA and folding index and increased psychotic symptom severity, further supporting the role of the region in schizophrenia. Notably, we observed an opposite directionality of effect for H3-HK methylation WG PRSs and gene-set PRSs on entorhinal SA, which we hypothesize could be due to differential action of the pathway SNPs versus the WG SNPs.

Interestingly, the dendritic spine and PSM gene sets were not associated with any of the phenotypes that were investigated. Both cellular components have been linked to schizophrenia previously and are closely related to the other biologically relevant pathways investigated here (i.e., PSD, axon gene sets) (60,61). This may indicate that, while these gene sets are relevant in schizophrenia, we may need additional information [e.g., interaction with other relevant biological pathways or investigation of copy number variants in relation to these cellular components (62)] to identify associations with these reported PLE and structural brain features. Despite correlation analyses of PRSs across participants revealing a strong positive correlation between PSD- and PSM-pathway PRSs (*r* = 0.63, *p* ≤ .01), this did not translate to similarly strong associations within the main analyses.

All PLEs, as well as distress, were significantly associated with WG PRSs (excluding SNPs in each gene set), as expected. This indicates that the PRSs generated from the schizophrenia GWAS were able to predict schizophrenia-related traits and therefore are a valuable tool. In addition, a number of neuroimaging phenotypes were associated with WG PRSs (irrespective of which gene set–specific SNPs were removed prior to calculation), including white matter microstructure and cortical and subcortical volume regions (see Table S4 in the Supplement for list of regions). These areas have been associated with WG PRSs in previous studies, calculated both with the GWAS summary statistics utilized here and with those from older GWASs (63,64). These findings confirm previous results and provide additional evidence of an association between increased genetic risk for schizophrenia and disruptions in gray and white matter. However, the finding that WG PRSs (excluding SNPs in each gene set) more accurately predicted schizophrenia-relevant phenotypes over pathway PRSs for these measures at this stage qualifies our aim to address the disorder’s heterogeneity by selecting biologically relevant pathways. Future studies could incorporate additional factors such as expression-quantitative trait loci to PRSs to determine whether pathway-specific gene expression on brain tissue can better differentiate variation in specific phenotypes over nonpathway expression trends.

The current study has a number of strengths. We utilized the largest, most recent GWAS of schizophrenia with a large sample comprising reported-PLEs and neuroimaging datasets. Our findings were further tested utilizing permutation analysis, which indicated that results were not due to chance. Finally, we were able to identify associations that could be utilized in the future to identify stratified patient populations, potentially leading to earlier diagnosis and applied interventions.

Limitations include the investigation of these associations in a general population sample, in which participants are generally healthier and wealthier than the general population, as shown by Fry *et al.* (65). Due to the low number of ICD-10 diagnosed schizophrenia participants with reported-PLEs (*n* = 123) and imaging (*n* = 49) measures, we were unable to attempt a replication and highly encourage that this be done in larger cohorts. This may partially explain the low effect sizes we observed throughout, and it is expected that effect sizes will increase as clinical samples with available genetic and neuroimaging data increase in number. We are unable to generalize our findings to populations of non-European ancestry because both our GWAS and target samples consisted exclusively of individuals of European descent. However, efforts are continually being made to collect data from people of other ancestries, and the associations identified here could be explored in these cohorts.

A further limitation includes the use of the MHQ, which measures lifetime occurrence of PLEs rather than PLEs that occurred at a specific time point. Therefore, our results may differ from those obtained in samples of participants in other age groups. Furthermore, responses were self-reported, and it is unclear whether reported PLEs arose from normal experiences or were linked to a psychiatric disorder. The strong associations with schizophrenia for WG PRSs (excluding SNPs in each gene set) indicate that the items measure some psychosis-related features. However, although PLEs are a hallmark of schizophrenia, they may be shared by a number of mental health disorders and nonclinical phenotypes (66,67). As such, schizophrenia-implicated pathways may not fully capture the genetic basis for PLEs. A multidisorder study focused on the severity of PLEs encompassing pathways associated with other mental health disorders could disentangle potentially shared genetic pathways associated with PLEs.

The differential directionality of effect for a number of pathway PRSs and WG (excluding SNPs in each gene set) PRSs may signal the presence of other disorder-contributing factors which may have differential pathway and WG effects that were not controlled for here. Future studies could include a wider range of covariates encompassing both environmental and genomic factors, such as smoking status and methylation.

Lastly, several methodological limitations should be noted. Reference-based approaches, such as the pathway-of-interest selection employed here, may limit the strength of their findings (68) by lowering the threshold required for a pathway to be significantly associated with a phenotype. An omnibus study utilizing all available pathways could be useful in contextualizing the strength of these findings in relation to the larger pool of gene ontologies not previously associated with schizophrenia. Although FreeSurfer is a standard methodological approach to analyzing structural MRI data across psychiatry, it may have limitations compared with manual tracing approaches, especially concerning segmentation variability (69). Future studies could utilize a combination of image processing software, such as FreeSurfer and voxel-based morphometry, to validate their findings. Finally, window-based approaches for SNP-gene mapping are limited by their chosen arbitrary distances and may be less informative than methodologies using gene expression data. Ultimately, this study is intrinsically limited by its inclusion of only 5 biologically relevant pathways and a limited number and type of reported-PLEs and neuroimaging measures and the methods used to obtain these. Future studies could complement this investigation by diversifying these parameters, especially in the case of utilizing functional imaging measures, as well as by replicating our findings in other large cohorts.

The pathways investigated here were previously found to be implicated in schizophrenia in both animal and human studies (4,17,53). In this study, we identified structural neuroimaging and reported-PLEs phenotypes that were associated with biologically informed PRSs, and these were shown to be more strongly associated with neuroimaging phenotypes than WG PRSs. However, because associations were stronger overall for WG PRSs, our findings also indicate that genetic risk aggregated to biologically relevant pathways is not yet more informative than genome-wide risk, but it may be still of relevance to future studies attempting to address heterogeneity and stratify individuals by genetic risk.

## Supplementary Material

Supplementary Material

Supplementary Excel File 1

Supplementary Excel File 2

## References

[1] Owen M.J., Sawa A., Mortensen P.B. (2016). Schizophrenia. Lancet.

[2] Hilker R., Helenius D., Fagerlund B., Skytthe A., Christensen K., Werge T.M. (2018). Heritability of schizophrenia and schizophrenia spectrum based on the nationwide Danish twin register. Biol Psychiatry.

[3] Lam M., Chen C.Y., Li Z., Martin A.R., Bryois J., Ma X. (2019). Comparative genetic architectures of schizophrenia in East Asian and European populations. Nat Genet.

[4] Trubetskoy V., Pardiñas A.F., Qi T., Panagiotaropoulou G., Awasthi S., Bigdeli T.B. (2022). Mapping genomic loci implicates genes and synaptic biology in schizophrenia. Nature.

[5] O’dushlaine C., Rossin L., Lee P.H., Duncan L., Parikshak N.N., Newhouse S. (2015). Psychiatric genome-wide association study analyses implicate neuronal, immune and histone pathways [published correction appears in Nat Neurosci 2015;18:926] [published correction appears in Nat Neurosci 201;18:1861]. Nat Neurosci.

[6] Rampino A., Taurisano P., Fanelli G., Attrotto M., Torretta S., Antonucci L.A. (2017). A Polygenic Risk Score of glutamatergic SNPs associated with schizophrenia predicts attentional behavior and related brain activity in healthy humans. Eur Neuropsychopharmacol.

[7] Yao Y., Guo W., Zhang S., Yu H., Yan H., Zhang H. (2021). Cell type-specific and cross-population polygenic risk score analyses of MIR137 gene pathway in schizophrenia. iScience.

[8] Terwisscha Van Scheltinga A.F., Bakker S.C., van Haren N.E., Derks E.M., Buizer-Voskamp J.E., Boos H.B.M. (2013). Genetic schizophrenia risk variants jointly modulate total brain and white matter volume. Biol Psychiatry.

[9] Alnæs D., Kaufmann T., van der Meer D., Córdova-Palomera A., Rokicki J., Moberget T. (2019). Brain heterogeneity in schizophrenia and its association with polygenic risk [published correction appears in JAMA Psychiatry 2019;76:986]. JAMA Psychiatry.

[10] Grama S., Willcocks I., Hubert J.J., Pardiñas A.F., Legge S.E., Bracher-Smith M. (2020). Polygenic risk for schizophrenia and subcortical brain anatomy in the UK Biobank cohort. Transl Psychiatry.

[11] Barbu M.C., Zeng Y., Shen X., Cox S.R., Clarke T.K., Gibson J. (2019). Association of whole-genome and NETRIN1 signaling pathway-derived polygenic risk scores for major depressive disorder and white matter microstructure in the UK Biobank. Biol Psychiatry Cogn Neurosci Neuroimaging.

[12] Moyer C.E., Shelton M.A., Sweet R.A. (2015). Dendritic spine alterations in schizophrenia. Neurosci Lett.

[13] Föcking M., Lopez L.M., English J.A., Dicker P., Wolff A., Brindley E. (2015). Proteomic and genomic evidence implicates the postsynaptic density in schizophrenia. Mol Psychiatry.

[14] Shen E., Shulha H., Weng Z., Akbarian S. (2014). Regulation of histone H3K4 methylation in brain development and disease. Philos Trans R Soc Lond B Biol Sci.

[15] Sudlow C., Gallacher J., Allen N., Beral V., Burton P., Danesh J. (2015). UK Biobank: An open access resource for identifying the causes of a wide range of complex diseases of middle and old age. PLoS Med.

[16] Global Core Biodata Resources The gene ontology resource. http://geneontology.org/.

[17] Gupta S., Kim S.Y., Artis S., Molfese D.L., Schumacher A., Sweatt J.D. (2010). Histone methylation regulates memory formation. J Neurosci.

[18] Zhu Y., Wang S., Gong X., Edmiston E.K., Zhong S., Li C. (2021). Associations between hemispheric asymmetry and schizophrenia-related risk genes in people with schizophrenia and people at a genetic high risk of schizophrenia. Br J Psychiatry.

[19] Bycroft C., Freeman C., Petkova D., Band G., Elliott L.T., Sharp K. (2018). The UK biobank resource with deep phenotyping and genomic data. Nature.

[20] Choi S.W., Mak T.S., O’Reilly P.F. (2020). Tutorial: A guide to performing polygenic risk score analyses. Nat Protoc.

[21] Smith B.H., Campbell A., Linksted P., Fitzpatrick B., Jackson C., Kerr S.M. (2013). Cohort profile: Generation Scotland: Scottish Family Health study (GS:SFHS). The study, its participants and their potential for genetic research on health and illness. Int J Epidemiol.

[22] Wang K., Li M., Hakonarson H. (2010). ANNOVAR: Functional annotation of genetic variants from high-throughput sequencing data. Nucleic Acids Res.

[23] Euesden J., Lewis C.M., O’Reilly P.F. (2015). PRSice: Polygenic Risk Score software. Bioinformatics.

[24] Schoorl J., Barbu M.C., Shen X., Harris M.R., Adams M.J., Whalley H.C. (2021). Grey and white matter associations of psychotic-like experiences in a general population sample (UK Biobank). Transl Psychiatry.

[25] Alloza C., Blesa-Cábez M., Bastin M.E., Madole J.W., Buchanan C.R., Janssen J. (2020). Psychotic-like experiences, polygenic risk scores for schizophrenia, and structural properties of the salience, default mode, and central-executive networks in healthy participants from UK Biobank. Transl Psychiatry.

[26] Bosma M.J., Cox S.R., Ziermans T., Buchanan C.R., Shen X., Tucker-Drob E.M. (2023). White matter, cognition and psychotic-like experiences in UK Biobank. Psychol Med.

[27] Alfaro-Almagro F., Jenkinson M., Bangerter N.K., Andersson J.L.R., Griffanti L., Douaud G. (2018). Image processing and quality control for the first 10,000 brain imaging datasets from UK Biobank. Neuroimage.

[28] Smith S.M., Alfaro-Almagro F., Miller K.L. UK Biobank brain imaging documentation version 1.9. https://biobank.ctsu.ox.ac.uk/crystal/crystal/docs/brain_mri.pdf.

[29] Shen X., Adams M.J., Ritakari T.E., Cox S.R., McIntosh A.M., Whalley H.C. (2019). White matter microstructure and its relation to longitudinal measures of depressive symptoms in mid- and late life. Biol Psychiatry.

[30] Shen X., Reus L.M., Cox S.R., Adams M.J., Liewald D.C., Bastin M.E. (2017). Subcortical volume and white matter integrity abnormalities in major depressive disorder: Findings from UK Biobank imaging data. Sci Rep.

[31] Alexander B., Loh W.Y., Matthews L.G., Murray A.L., Adamson C., Beare R. (2019). Desikan-Killiany-Tourville Atlas compatible version of m-CRIB neonatal parcellated whole brain atlas: The m-Crib 2.0. Front Neurosci.

[32] Elliott L.T., Sharp K., Alfaro-Almagro F., Shi S., Miller K.L., Douaud G. (2018). Genome-wide association studies of brain imaging phenotypes in UK Biobank. Nature.

[33] Fischl B., Salat D.H., Busa E., Albert M., Dieterich M., Haselgrove C. (2002). Whole brain segmentation: Automated labeling of neuroanatomical structures in the human brain. Neuron.

[34] O’Connell K.S., Sønderby I.E., Frei O., Van Der Meer D., Athanasiu L., Smeland O.B. (2021). Association between complement component 4A expression, cognitive performance and brain imaging measures in UK Biobank. Psychol Med.

[35] Green C., Stolicyn A., Harris M.A., Shen X., Romaniuk L., Barbu M.C. (2021). Hair glucocorticoids are associated with childhood adversity, depressive symptoms and reduced global and lobar grey matter in Generation Scotland. Transl Psychiatry.

[36] Benjamini Y., Hochberg Y. (1995). Controlling the false discovery rate: A practical and powerful approach to multiple testing. J R Stat Soc Series B Stat Methodol.

[37] Cabrera C.P., Navarro P., Huffman J.E., Wright A.F., Hayward C., Campbell H. (2012). Uncovering networks from genome-wide association studies via circular genomic permutation. G3 (Bethesda).

[38] Bayés A., Van De Lagemaat L.N., Collins M.O., Croning M.D.R., Whittle I.R., Choudhary J.S., Grant S.G. (2011). Characterization of the proteome, diseases and evolution of the human postsynaptic density. Nat Neurosci.

[39] Sorokina O., Mclean C., Croning M.D.R., Heil K.F., Wysocka E., He X. (2021). A unified resource and configurable model of the synapse proteome and its role in disease [published correction appears in Sci Rep 2021;11:16240]. Sci Rep.

[40] Fromer M., Pocklington A.J., Kavanagh D.H., Williams H.J., Dwyer S., Gormley P. (2014). De novo mutations in schizophrenia implicate synaptic networks. Nature.

[41] Purcell S.M., Moran J.L., Fromer M., Ruderfer D., Solovieff N., Roussos P. (2014). A polygenic burden of rare disruptive mutations in schizophrenia. Nature.

[42] Skene N.G., Roy M., Grant S.G. (2017). A genomic lifespan program that reorganises the young adult brain is targeted in schizophrenia. eLife.

[43] Nithianantharajah J., Komiyama N.H., McKechanie A., Johnstone M., Blackwood D.H., Clair D.S. (2012). Synaptic scaffold evolution generated components of vertebrate cognitive complexity. Nat Neurosci.

[44] Bersani F.S., Minichino A., Fojanesi M., Gallo M., Maglio G., Valeriani G. (2014). Cingulate cortex in schizophrenia: Its relation with negative symptoms and psychotic onset. A review study. Eur Rev Med Pharmacol Sci.

[45] Wang L., Hosakere M., Trein J.C.L., Miller A., Ratnanather J.T., Barch D.M. (2007). Abnormalities of cingulate gyrus neuroanatomy in schizophrenia [published correction appears in Schizophr Res 2007;94:380. Schizophr Res.

[46] Neilson E., Shen X., Cox S.R., Clarke T.K., Wigmore E.M., Gibson J. (2019). Impact of polygenic risk for schizophrenia on cortical structure in UK Biobank. Biol Psychiatry.

[47] Zhu X., Ward J., Cullen B., Lyall D.M., Strawbridge R.J., Smith D.J., Lyall L.M. (2021). Polygenic risk for schizophrenia, brain structure, and environmental risk in UK Biobank. Schizophr Bull Open.

[48] Wang Z., Li P., Wu T., Zhu S., Deng L., Cui G. (2018). Axon guidance pathway genes are associated with schizophrenia risk. Exp Ther Med.

[49] Mukai J., Tamura M., Fénelon K., Rosen A.M., Spellman T.J., Kang R. (2015). Molecular substrates of altered axonal growth and brain connectivity in a mouse model of schizophrenia. Neuron.

[50] Lebel C., Deoni S. (2018). The development of brain white matter microstructure. Neuroimage.

[51] Van Hoesen G.W., Augustinack J.C., Dierking J., Redman S.J., Thangavel R. (2000). The parahippocampal gyrus in Alzheimer’s disease. Clinical and preclinical neuroanatomical correlates. Ann N Y Acad Sci.

[52] McIntosh A.M., Muñoz Maniega S., Lymer G.K., McKirdy J., Hall J., Sussmann J.E.D. (2008). White matter tractography in bipolar disorder and schizophrenia. Biol Psychiatry.

[53] Zhu X., Ward J., Cullen B., Lyall D.M., Strawbridge R.J., Lyall L.M. (2021). Phenotypic and genetic associations between anhedonia and brain structure in UK Biobank. Transl Psychiatry.

[54] Greer E.L., Shi Y. (2012). Histone methylation: A dynamic mark in health, disease and inheritance. Nat Rev Genet.

[55] Gavin D.P., Sharma R.P. (2010). Histone modifications, DNA methylation, and Schizophrenia. Neurosci Biobehav Rev.

[56] Montano C., Taub M.A., Jaffe A., Briem E., Feinberg J.I., Trygvadottir R. (2016). Association of DNA methylation differences with schizophrenia in an epigenome-wide association study. JAMA Psychiatry.

[57] Huang H.S., Matevossian A., Whittle C., Se Y.K., Schumacher A., Baker S.P., Akbarian S. (2007). Prefrontal dysfunction in schizophrenia involves mixed-lineage leukemia 1-regulated histone methylation at GABAergic gene promoters. J Neurosci.

[58] Schultz C.C., Koch K., Wagner G., Roebel M., Schachtzabel C., Nenadic I. (2010). Psychopathological correlates of the entorhinal cortical shape in schizophrenia. Eur Arch Psychiatry Clin Neurosci.

[59] Sumiyoshi T., Tsunoda M., Uehara T., Tanaka K., Itoh H., Sumiyoshi C., Kurachi M. (2004). Enhanced locomotor activity in rats with excitotoxic lesions of the entorhinal cortex, a neurodevelopmental animal model of schizophrenia: Behavioral and in vivo microdialysis studies. Neurosci Lett.

[60] Glausier J.R., Lewis D.A. (2013). Dendritic spine pathology in schizophrenia. Neuroscience.

[61] De Bartolomeis A., Latte G., Tomasetti C., Iasevoli F. (2014). Glutamatergic postsynaptic density protein dysfunctions in synaptic plasticity and dendritic spines morphology: Relevance to schizophrenia and other behavioral disorders pathophysiology, and implications for novel therapeutic approaches. Mol Neurobiol.

[62] Kirov G., Pocklington A.J., Holmans P., Ivanov D., Ikeda M., Ruderfer D. (2012). De novo CNV analysis implicates specific abnormalities of postsynaptic signalling complexes in the pathogenesis of schizophrenia. Mol Psychiatry.

[63] van der Merwe C., Passchier R., Mufford M., Ramesar R., Dalvie S., Stein D.J. (2019). Polygenic risk for schizophrenia and associated brain structural changes: A systematic review. Compr Psychiatry.

[64] Stauffer E.M., Bethlehem R.A.I., Warrier V., Murray G.K., Romero-Garcia R., Seidlitz J., Bullmore E.T. (2021). Grey and white matter microstructure is associated with polygenic risk for schizophrenia. Mol Psychiatry.

[65] Fry A., Littlejohns T.J., Sudlow C., Doherty N., Adamska L., Sprosen T. (2017). Comparison of sociodemographic and health-related characteristics of UK Biobank participants with those of the general population. Am J Epidemiol.

[66] Kelleher I., Cannon M. (2011). Psychotic-like experiences in the general population: Characterizing a high-risk group for psychosis. Psychol Med.

[67] Legge S.E., Jones H.J., Kendall K.M., Pardiñas A.F., Menzies G., Bracher-Smith M. (2019). Association of genetic liability to psychotic experiences with neuropsychotic disorders and traits. JAMA Psychiatry.

[68] Pergola G., Penzel N., Sportelli L., Bertolino A. (2022). Lessons learned from parsing genetic risk for schizophrenia into biological pathways [published online Oct 28]. Biol Psychiatry.

[69] Morey R.A., Petty C.M., Xu Y., Hayes J.P., Wagner H.R., Lewis D.V. (2009). A comparison of automated segmentation and manual tracing for quantifying hippocampal and amygdala volumes. Neuroimage.

