# Supplementary material for: Pathway-Based Polygenic Risk Scores for Schizophrenia and Associations With Reported Psychotic-like Experiences and Neuroimaging Phenotypes in the UK Biobank
Source: Biol Psychiatry Glob Open Sci. 2023 Mar 25;3(4):814–23. doi: 10.1016/j.bpsgos.2023.03.004 (PMC10593950; doi:10.1016/j.bpsgos.2023.03.004)
Supplement: Supplementary Material [file mmc1.pdf]

SUPPLEMENTARY INFORMATION

Pathway-based Polygenic Risk Scores for Schizophrenia and Associations With Reported Psychotic-like Experiences and Neuroimaging Phenotypes in UK Biobank

Barbu *et al.*

Supplementary Materials

SNP QC

The following quality checks (QC) were performed based on the protocol by Choi et al. (1) https://choishingwan.github.io/PRS-Tutorial/base/): removing SNPs with a minor allele frequency (MAF) < 0.01, and imputation information score < 0.8; removing duplicated and ambiguous SNPs; and ensuring that there were no overlapping samples with the target dataset (UKB). This resulted in a final dataset containing N=5,899,138 SNPs. QC in UKB was performed based on the same protocol (1) (https://choishingwan.github.io/PRS-Tutorial/base/): removing SNPs with a MAF < 0.01, Hardy-Weinberg Equilibrium (HWE) < $1x10^{-6}$, SNPs that are missing in a high proportion of participants (<0.02), highly-correlated SNPs (r^2=0.25); and removing individuals that have a high rate of genotype missingness (0.02) and that contain high heterozygosity rates (> 3 SDs from the mean).

SNP annotation and PRS calculation

ANNOVAR was utilised to annotate SNPs in the base dataset, if they were located between 20 kb upstream or downstream of the transcription start and end sites, respectively (2). Gene-set PRSs for each individual in UKB were computed using PRSice at 5 p-value thresholds (0.01, 0.05, 0.1, 0.5, and 1) by summing the number of risk alleles weighted by the strength of association with schizophrenia in the base dataset. Each gene-set PRS had its respective whole-genome PRS (excluding SNPs in each gene-set) (WG PRS). PRSs were created with clumping-based pruning of SNPs in linkage disequilibrium (r2=0.25, 500-kb window). SNPs within the extended MHC locus (chr6:25 Mb–35Mb) were excluded due to high LD in the region.

MHQ questions utilised for reported-PLE phenotypes

"Did you ever believe that there was an unjust plot going on to harm you or to have people follow you, and which your family and friends did not believe existed?"

"Did you ever believe that a strange force was trying to communicate directly with you by sending special signs or signals that you could understand but that no one else could understand (for example through the radio or television)?"

"Did you ever hear things that other people said did not exist, like strange voices coming from inside your head talking to you or about you, or voices coming out of the air when there was no one around?"

"Did you ever see something that wasn't really there that other people could not see?".

UK Biobank MRI protocols

T1 weighted and diffusion (DTI) MRI images were acquired using a Siemens Skyra 3 T scanner with a standard Siemens 32-channel RF receive head coil. Magnetic resonance imaging (MRI) acquisition, pre-processing, and QC for all structural phenotypes were performed utilising standardized protocols in UKB and are described in detail elsewhere (https://biobank.ctsu.ox.ac.uk/crystal/crystal/docs/brain_mri.pdf) (3, 4). Exclusion criteria comprised removal of scans with severe normalization problems by the UKB.

Permutation analysis

First, for significant gene-set associations, circular genomic permutation was applied, by placing all SNPs in the genome (excluding those pertaining to each of the gene-sets demonstrating significance) in a circular genome, based on their location (5). One thousand SNP lists with the same set size as each of these gene-sets were then permuted. Then, we created 1,000 PRSs based on these SNP lists, that were then included in regression models for the phenotypes that were originally identified to be significantly associated with the gene-set PRSs. Significance was then determined by comparing t-values from the real associations with those derived from the permuted regression models. The permutation p-value was calculated by observing the position of the real t-values in the list of permuted t-values and dividing the position by the number of permutations (N=1,000).

Biological pathway information

The axon pathway represents the part of the neuron that conducts nerve impulses away from the cell bodies; electrical signals are then received by other neurons. The pathway is classified as a cellular component on the Gene Ontology database (GO term: 0030424 http://amigo.geneontology.org/amigo/term/GO:0030424).

The histone H3-K4 methylation pathway involves the modification of the H3 histone by addition of one or more methyl groups to lysine at position 4 of the histone. The pathway is classified as a biological process in Gene Ontology (GO term: 0051568; http://amigo.geneontology.org/amigo/term/GO:0051568). The pathway plays a role in brain development and cell differentiation. Dysregulated H3-K4 methylation has been associated with schizophrenia and autism (6).

The dendritic spine pathway is classified as a cellular component, comprises a membranous protrusion from dendrites that receives input from a presynapse, and can have variable spine

morphology (GO term: 0043197; http://amigo.geneontology.org/amigo/term/GO:0043197). Dendritic spine impairments are present in schizophrenia (7).

The postsynaptic density pathway is a cellular component comprising an electron-dense protein network that is situated within and adjacent to the postsynaptic membrane of an asymmetric synapse (GO term: 0014069; http://amigo.geneontology.org/amigo/term/GO:0014069). The postsynaptic membrane comprises a specialised area of the membrane localised to the nerve ending and separated by the synaptic cleft; neurotransmitters cross this and transmit signals to the postsynaptic membrane (GO term: 0045211; http://amigo.geneontology.org/amigo/term/GO:0045211). Both have been previously implicated in schizophrenia, in genomic- and proteomic-specific studies (8).

	**Postsynaptic density**	**Postsynaptic membrane**	**Dendritic spine**	**Histone He-K4 methylation**	**Axon**
**N pathway genes in Trubetskoy et al. (2022)** (9)	312/332	264/298	178/185	56/62	613/653
**N SNPs**	180,704	181,555	84,601	10,732	257,383

Supplementary Table 1. The number of genes in each pathway and those that are included in the discovery genome-wide association study (GWAS); the number of SNPs in each pathway that are present in the discovery GWAS.

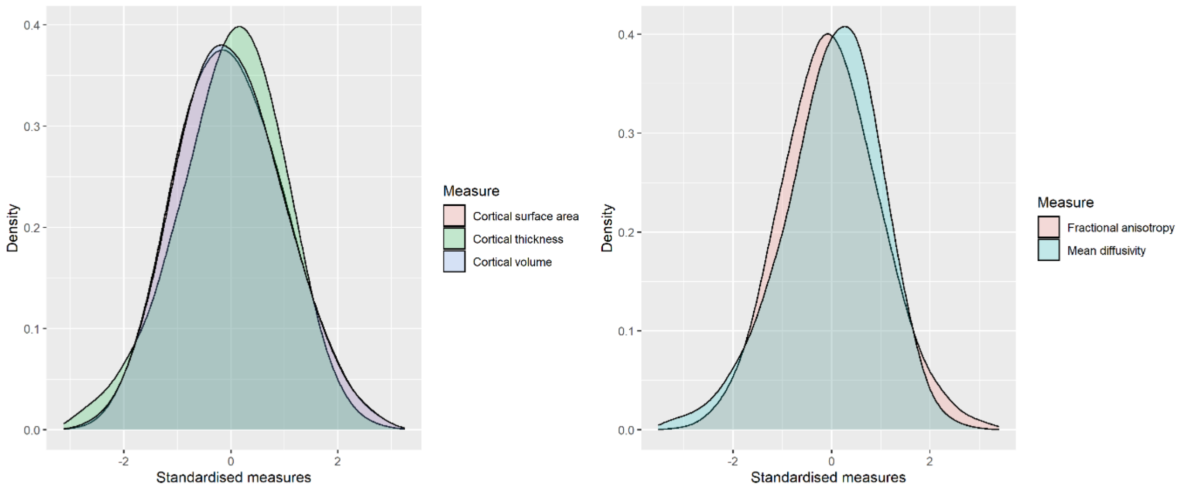

Supplementary Figure 1. Density plots of global measures of cortical regions (surface area, thickness, volume) and white matter microstructure (fractional anisotropy, mean diffusivity). The x-axis indicates standardized measures for each neuroimaging phenotype, and the y-axis represents the distribution density.

Imaging modality	Variable name	UKB variable ID		
Cortical regions	**Global**	Sum of all individual measures below	Sum of all individual measures below	Sum of all individual measures below
		**Area**	**Cortical volume**	**Mean thickness**
	**Frontal lobe**	Sum of the below	Sum of the below	Sum of the below
	Superior frontal gyrus	27168, 27261	27230, 27323	27199, 27292
	Rostral middle frontal	27167, 27260	27229, 27322	27198, 27291
	Caudal middle frontal	27144, 27237	27206, 27299	27175, 27268
	Pars orbitalis	27159, 27252	27221, 27314	27190, 27283
	Pars triangularis	27160, 27253	27222, 27315	27191, 27284
	Pars opercularis	27158, 27251	27220, 27313	27189, 27282
	Lateral orbitofrontal	27152, 27245	27214, 27307	27183, 27276
	Medial orbitofrontal	27154, 27247	27216, 27309	27185, 27278
	Precentral gyrus	27164, 27257	27226, 27319	27195, 27288
	Paracentral cortex	27157, 27250	27219, 27312	27186, 27281
	**Temporal lobe**	Sum of the below	Sum of the below	Sum of the below
	Insula	27173, 27266	27235, 27328	27204, 27204
	Superior temporal	27170, 27263	27232, 27325	27201, 27294
	Transverse temporal	27172, 27265	27234, 27327	27203, 27296
	Middle temporal gyrus	27155, 27248	27217, 27310	27186, 27186
	Inferior temporal gyrus	27149, 27242	27211, 27304	27180, 27273
	Fusiform	27147, 27240	27209, 27302	27178, 27271
	Parahippocampal	27156, 27249	27218, 27311	27187, 27280
	Entorhinal	27146, 27239	27208, 27301	27177, 27270
	**Parietal lobe**	Sum of the below	Sum of the below	Sum of the below
	Postcentral gyrus	27162, 27255	27224, 27317	27193, 27286
	Paracentral cortex	27157, 27250	27219, 27312	27186, 27281
	Superior parietal cortex	27169, 27262	27231, 27324	27200, 27293
	Inferior parietal cortex	27148, 27241	27210, 27303	27179, 27272
	Supramarginal gyrus	27171, 27264	27233, 27326	27202, 27295
	Precuneus	27165, 27258	27227, 27320	27196, 27289
	**Occipital lobe**	Sum of the below	Sum of the below	Sum of the below
	Lateral occipital cortex	27151, 27244	27213, 27306	27182, 27275
	Cuneus	27145, 27238	27207, 27300	27176, 27269
	Pericalcarine cortex	27161, 27254	27223, 27316	27192, 27285
	Lingual gyrus	27153, 27246	27215, 27308	27184, 27277
	**Cingulate lobe**	Sum of the below	Sum of the below	Sum of the below
	Rostral anterior cingulate	27166, 27259	27228, 27321	27197, 27290

	Caudal anterior cingulate	27143, 27236	27205, 27298	27174, 27267
	Posterior cingulate cortex	27163, 27256	27225, 27225	27194, 27287
	Lingual gyrus	27153, 27246	27215, 27308	27184, 27277
**DTI measures**	**Global measure (PCA)**	**FA**	**MD**	
	**Projection fibres**	PCA of the below	PCA of the below	
	Forceps major	25498	25525	
	Forceps minor	25499	25526	
	Corticospinal tract	25496, 25497	25523, 25524	
	Acoustic radiation	25488, 25489	25515, 25516	
	Medial lemniscus	25505, 25506	25532, 25533	
	Middle cerebellar peduncle	25504	25531	
	**Association fibres**	PCA of the below	PCA of the below	
	Inferior fronto-occipital fasciculus	25500, 25501	25527, 25528	
	Uncinate fasciculus	25513, 25514	25540, 25541	
	Cingulum bundle (gyrus)	25492, 25493	25519, 25520	
	Cingulum bundle (parahippocampal)	25494, 25495	25521, 25522	
	Superior longitudinal fasciculus	25509, 25510	25536, 25537	
	Inferior longitudinal fasciculus	25502, 25503	25529, 25530	
	**Thalamic radiations**	PCA of the below	PCA of the below	
	Superior thalamic radiations	25511, 25512	25538, 25539	
	Posterior thalamic radiations	25507, 25508	25534, 25535	
	Anterior thalamic radiations	25490, 25491	25517, 25518	
**Subcortical volumes**	Thalamus	25011, 25012		
	Caudate	25013, 25014		
	Putamen	25015, 25016		
	Pallidum	25017, 25018		
	Hippocampus	25019, 25020		
	Amygdala	25021, 25022		
	Accumbens	25023, 25024		
	Intracranial volume	Sum of: 25005, 25007, 25003		

Supplementary Table 2. Imaging modalities investigated. Where two IDs are given, the first corresponds to the left hemisphere and the second to the right hemisphere.

Measure name	Number of measures FDR was applied for	Measure name	Number of measures FDR was applied for
**Bilateral**		**Unilateral**	
Cortical Area Individual ROIs	31 x 5 pathways x 20 pathway p-value thresholds	Cortical Area Lobar	5 x 5 pathways x 20 pathway p-value thresholds
Cortical Thickness Individual ROIs	31 x 5 pathways x 20 pathway p-value thresholds	Cortical Thickness Lobar	5 x 5 pathways x 20 pathway p-value thresholds
Cortical Volume Individual ROIs	31 x 5 pathways x 20 pathway p-value thresholds	Cortical Volume Lobar	5 x 5 pathways x 20 pathway p-value thresholds
FA Individual ROIs	12 x 5 pathways x 20 pathway p-value thresholds	FA Individual	3 x 5 pathways x 20 pathway p-value thresholds
MD Individual ROIs	12 x 5 pathways x 20 pathway p-value thresholds	MD Individual	3 x 5 pathways x 20 pathway p-value thresholds
		FA tract bundles	3 x 5 pathways x 20 pathway p-value thresholds
		MD tract bundles	3 x 5 pathways x 20 pathway p-value thresholds

Supplementary Table 3. Procedure for False Discovery Rate (FDR) corrections.

Measure	WG PRS	Standardized effect size	SE	z-value	p-value	p-value (FDR)
**FA**						
Global	Axon	-0.0156	0.0058	-2.6965	0.007011	**0.03032**
	Dendritic spine	-0.0181	0.0058	-3.1376	0.001705	**0.006201**
	Histone	-0.0178	0.0058	-3.0959	0.001964	**0.007724**
	Post-synaptic density	-0.0175	0.0058	-3.0391	0.002375	**0.009501**
	Post-synaptic membrane	-0.019	0.0058	-3.2935	0.000991	**0.004644**
Association fibres	Axon	-0.0179	0.0058	-3.1029	0.001918	**0.01918**
	Dendritic spine	-0.0195	0.0057	-3.4004	0.000674	**0.006143**
	Histone	-0.0194	0.0057	-3.389	0.000702	**0.007024**
	Post-synaptic density	-0.0189	0.0057	-3.2951	0.000985	**0.008798**
	Post-synaptic membrane	-0.0207	0.0057	-3.6198	0.000295	**0.002965**
Thalamic radiations	Axon	-0.0143	0.0058	-2.464	0.01375	**0.03584**
	Dendritic spine	-0.0182	0.0058	-3.1411	0.001685	**0.006201**
	Histone	-0.0175	0.0058	-3.0224	0.00251	**0.007724**
	Post-synaptic density	-0.0165	0.0058	-2.8498	0.004378	**0.01347**
	Post-synaptic membrane	-0.0178	0.0058	-3.0657	0.002174	**0.006689**
Cingulate gyrus	Axon	-0.0154	0.0045	-3.3924	0.000694	**0.03441**
	Dendritic spine	-0.0146	0.0045	-3.23	0.001239	**0.01928**
	Histone	-0.0151	0.0045	-3.3446	0.000825	**0.02546**
	Post-synaptic density	-0.0158	0.0045	-3.4903	0.000483	**0.02416**
	Post-synaptic membrane	-0.0153	0.0045	-3.3895	0.000701	**0.01484**
Anterior thalamic radiations	Dendritic spine	-0.0169	0.0055	-3.0961	0.001963	**0.02307**
	Histone	-0.0167	0.0055	-3.0726	0.002124	**0.02845**
	Post-synaptic density	-0.0161	0.0055	-2.9418	0.003266	**0.03882**
	Post-synaptic membrane	-0.0166	0.0055	-3.0519	0.002276	**0.01897**
Posterior thalamic radiations	Axon	-0.0159	0.0054	-2.941	0.003274	**0.02455**
	Post-synaptic membrane	-0.0152	0.0054	-2.8246	0.004737	**0.03089**

Inferior longitudinal fasciculus	Dendritic spine	-0.0162	0.0054	-3.0284	0.002461	**0.02307**
	Histone	-0.0151	0.0053	-2.8174	0.004845	**0.04275**
	Post-synaptic membrane	-0.0168	0.0054	-3.1484	0.001643	**0.01484**
Inferior fronto-occipital fasciculus	Dendritic spine	-0.0149	0.0053	-2.7948	0.005196	**0.03543**
	Post-synaptic membrane	-0.0157	0.0053	-2.9325	0.003365	**0.02404**
**MD**						
Global	Axon	0.0156	0.0054	2.8938	0.003809	**0.02541**
	Dendritic spine	0.0172	0.0054	3.2173	0.001295	**0.01127**
	Histone	0.0156	0.0053	2.9202	0.003501	**0.02673**
	Post-synaptic density	0.0153	0.0054	2.8484	0.004397	**0.03958**
	Post-synaptic membrane	0.0161	0.0053	3.0072	0.002639	**0.02165**
Association fibres	Dendritic spine	0.0142	0.0055	2.5677	0.01024	**0.03414**
	Post-synaptic membrane	0.0138	0.0055	2.5056	0.01223	**0.04213**
Projection fibres	Dendritic spine	0.0137	0.0056	2.4573	0.014	**0.04001**
Thalamic radiations	Axon	0.0154	0.0051	3.0215	0.002517	**0.02541**
	Dendritic spine	0.0166	0.0051	3.2619	0.001108	**0.01127**
	Histone	0.0149	0.0051	2.9311	0.00338	**0.02673**
	Post-synaptic density	0.0146	0.0051	2.8731	0.004068	**0.03958**
	Post-synaptic membrane	0.0152	0.0051	2.9861	0.002828	**0.02165**
Corticospinal tract	Axon	0.02	0.0054	3.7026	0.000214	**0.02269**
	Dendritic spine	0.0217	0.0054	4.0388	5.39E-05	**0.005314**
	Histone	0.0204	0.0054	3.8135	0.000137	**0.01046**
	Post-synaptic density	0.0209	0.0054	3.8802	0.000105	**0.01126**
	Post-synaptic membrane	0.0207	0.0054	3.8515	0.000118	**0.008926**
Anterior thalamic radiations	Dendritic spine	0.0153	0.005	3.0708	0.002137	**0.03683**
Superior thalamic radiations	Dendritic spine	0.0135	0.0049	2.7559	0.005858	**0.04333**
Posterior thalamic radiations	Dendritic spine	0.0134	0.005	2.6544	0.00795	**0.04969**

Inferior longitudinal fasciculus	Dendritic spine	0.0148	0.0052	2.8324	0.004624	**0.04101**
Cingulate gyrus	Dendritic spine	0.0154	0.0053	2.9199	0.003504	**0.04101**
**Subcortical Volume**						
Thalamus	Axon	-0.0215	0.0047	-4.5996	4.25E-06	**8.50E-05**
	Dendritic spine	-0.0225	0.0047	-4.8301	1.37E-06	**3.66E-05**
	Histone	-0.0202	0.0046	-4.3495	1.37E-05	**0.000365**
	Post-synaptic density	-0.0193	0.0047	-4.1473	3.37E-05	**0.0009**
	Post-synaptic membrane	-0.022	0.0046	-4.7262	2.30E-06	**6.13E-05**
Accumbens	Axon	-0.0145	0.0042	-3.4372	0.000589	**0.005231**
	Dendritic spine	-0.0137	0.0042	-3.2758	0.001055	**0.01055**
	Histone	-0.0127	0.0042	-3.0325	0.002428	**0.02158**
	Post-synaptic density	-0.0125	0.0042	-2.9765	0.002918	**0.03204**
	Post-synaptic membrane	-0.0142	0.0042	-3.3867	0.000708	**0.007083**
**Cortical Volume**						
Medial orbitofrontal cortex	Axon	-0.0159	0.0047	-3.3953	0.000687	**0.01773**
	Dendritic spine	-0.0187	0.0047	-4.0249	5.71E-05	**0.002952**
	Histone	-0.0166	0.0046	-3.5779	0.000347	**0.01792**
	Post-synaptic density	-0.0155	0.0047	-3.3302	0.000869	**0.02693**
	Post-synaptic membrane	-0.016	0.0047	-3.4367	0.00059	**0.02505**
Superior temporal gyrus	Axon	-0.019	0.0047	-4.0672	4.77E-05	**0.003698**
	Dendritic spine	-0.0203	0.0047	-4.3538	1.34E-05	**0.000832**
	Histone	-0.0183	0.0047	-3.9308	8.49E-05	**0.005261**
	Post-synaptic density	-0.0178	0.0047	-3.8112	0.000139	**0.008593**
	Post-synaptic membrane	-0.0187	0.0047	-4.0146	5.97E-05	**0.004627**
**Cortical Surface Area**						
Superior temporal gyrus	Dendritic spine	-0.0163	0.0045	-3.5924	0.000328	**0.02544**
	Histone	-0.0146	0.0045	-3.2304	0.001238	**0.04796**
	Post-synaptic membrane	-0.0161	0.0045	-3.5729	0.000354	**0.02234**

Supplementary Table 4. Neuroimaging phenotypes associated with whole-genome PRS (at <0.1 SNP significance threshold) (excluding SNPs in each gene-set).

Supplementary References

1. Choi SW, Mak TSH, O'Reilly PF. Tutorial: a guide to performing polygenic risk score analyses. Nat Protoc. 2020 Sep 1;15(9):2759–72.
2. Wang K, Li M, Hakonarson H. ANNOVAR: functional annotation of genetic variants from high-throughput sequencing data. Nucleic Acids Res [Internet]. 2010 Sep 1 [cited 2022 Apr 26];38(16):e164–e164. Available from: https://academic.oup.com/nar/article/38/16/e164/1749458
3. Alfaro-Almagro F, Jenkinson M, Bangerter NK, Andersson JLR, Griffanti L, Douaud G, et al. Image processing and Quality Control for the first 10,000 brain imaging datasets from UK Biobank. Neuroimage [Internet]. 2018 Feb 1 [cited 2022 Apr 25];166:400–24. Available from: https://pubmed.ncbi.nlm.nih.gov/29079522/
4. Smith SM, Alfaro-Almagro F, Miller KL. UK Biobank Brain Imaging Documentation UK Biobank Brain Imaging Documentation Contributors to UK Biobank Brain Imaging. [cited 2022 Apr 25]; Available from: http://www.ukbiobank.ac.uk
5. Cabrera CP, Navarro P, Huffman JE, Wright AF, Hayward C, Campbell H, et al. Uncovering networks from genome-wide association studies via circular genomic permutation. G3 (Bethesda) [Internet]. 2012 Sep [cited 2022 Apr 26];2(9):1067–75. Available from: https://pubmed.ncbi.nlm.nih.gov/22973544/
6. Shen E, Shulha H, Weng Z, Akbarian S. Regulation of histone H3K4 methylation in brain development and disease. Philos Trans R Soc B Biol Sci [Internet]. 2014 [cited 2022 May 5];369(1652). Available from: https://royalsocietypublishing.org/doi/full/10.1098/rstb.2013.0514
7. Moyer CE, Shelton MA, Sweet RA. Dendritic spine alterations in schizophrenia. Neurosci Lett. 2015 Aug 5;601:46–53.
8. Föcking M, Lopez LM, English JA, Dicker P, Wolff A, Brindley E, et al. Proteomic and genomic evidence implicates the postsynaptic density in schizophrenia. Mol Psychiatry 2015 204 [Internet]. 2014 Jul 22 [cited 2022 May 5];20(4):424–32. Available from: https://www.nature.com/articles/mp201463
9. Trubetskoy V, Pardiñas AF, Qi T, Panagiotaropoulou G, Awasthi S, Bigdeli TB, et al. Mapping genomic loci implicates genes and synaptic biology in schizophrenia. Nat 2022 6047906 [Internet]. 2022 Apr 8 [cited 2022 Apr 22];604(7906):502–8. Available from: https://www.nature.com/articles/s41586-022-04434-5
